# Supplementary material for: Development of chemokine network inhibitors using combinatorial saturation mutagenesis
Source: Commun Biol. 2025 Apr 3;8:549. doi: 10.1038/s42003-025-07778-6 (PMC11969024; doi:10.1038/s42003-025-07778-6)
Supplement: Supplementary file 4 — Supplementary Data 2 [file 42003_2025_7778_MOESM4_ESM.zip › SupplementaryData3/scripts/xattune_ci.tar!/./script/analysis/JK/JK_PMA_ExactPvalues.docx]

Supplementary Table: Exact P values for cell migration experiments, comparisons
to control

| ID | Experiment components | Control | Number of biological replicates | P value |
| --- | --- | --- | --- | --- |
| ATHERO | base | HD2SCR | 12 | 9.55e-14 |
| ATHERO | ATHERO | HD2SCR | 12 | 1.00e+00 |
| ATHERO | HD2SCR | HD2SCR | 12 | NA |
| ATHERO | HD2 | HD2SCR | 12 | 1.04e-02 |
| ATHERO | HD2Y5W | HD2SCR | 12 | 8.04e-03 |
| ATHERO | HD2T6D | HD2SCR | 12 | 6.90e-01 |
| ATHERO | HD2T6W | HD2SCR | 12 | 9.78e-02 |
| ATHERO | HD2A9W | HD2SCR | 12 | 6.29e-01 |
| ATHERO | HD2L11I | HD2SCR | 12 | 2.46e-01 |
| ATHERO | HD2T12W | HD2SCR | 12 | 8.54e-01 |
| ATHERO | HD2T16C | HD2SCR | 12 | 4.25e-02 |
| ATHERO | HD2CM304 | HD2SCR | 12 | 3.32e-05 |
| ATHERO | HD2CM307 | HD2SCR | 12 | 4.04e-04 |
| ATHERO | HD2CM452 | HD2SCR | 12 | 4.00e-03 |
| ATHERO | HD2CM526 | HD2SCR | 12 | 1.70e-02 |
| ATHERO | HD2CM539 | HD2SCR | 12 | 8.94e-04 |
| ATHERO | EVA4 | HD2SCR | 12 | 9.58e-08 |
| INS | base | HD2SCR | 12 | 8.27e-10 |
| INS | INS | HD2SCR | 12 | 9.96e-01 |
| INS | HD2SCR | HD2SCR | 12 | NA |
| INS | HD2 | HD2SCR | 12 | 1.00e+00 |
| INS | HD2Y5W | HD2SCR | 12 | 5.26e-03 |
| INS | HD2T6D | HD2SCR | 12 | 4.39e-01 |
| INS | HD2T6W | HD2SCR | 12 | 1.72e-02 |
| INS | HD2L11I | HD2SCR | 12 | 4.43e-01 |
| INS | HD2T12W | HD2SCR | 12 | 9.52e-01 |
| INS | HD2T16C | HD2SCR | 12 | 9.90e-01 |
| INS | HD2CM304 | HD2SCR | 12 | 7.61e-04 |
| INS | HD2CM307 | HD2SCR | 12 | 1.22e-03 |
| INS | HD2CM452 | HD2SCR | 12 | 1.30e-02 |
| INS | HD2CM526 | HD2SCR | 12 | 6.42e-01 |
| INS | HD2CM539 | HD2SCR | 12 | 1.49e-02 |
| INS | EVA4 | HD2SCR | 12 | 8.65e-02 |
| RA | base | HD2SCR | 9 | 1.36e-07 |
| RA | RA | HD2SCR | 12 | 1.00e+00 |
| RA | HD2SCR | HD2SCR | 12 | NA |
| RA | HD2 | HD2SCR | 12 | 9.81e-01 |
| RA | HD2Y5W | HD2SCR | 12 | 7.67e-02 |
| RA | HD2T6D | HD2SCR | 12 | 3.85e-01 |
| RA | HD2T6W | HD2SCR | 12 | 9.97e-01 |
| RA | HD2L11I | HD2SCR | 12 | 6.21e-01 |
| RA | HD2T12W | HD2SCR | 12 | 5.69e-01 |
| RA | HD2T16C | HD2SCR | 9 | 2.62e-03 |
| RA | HD2CM304 | HD2SCR | 12 | 1.71e-05 |
| RA | HD2CM307 | HD2SCR | 12 | 1.75e-02 |
| RA | HD2CM452 | HD2SCR | 12 | 2.01e-03 |
| RA | HD2CM526 | HD2SCR | 12 | 1.00e+00 |
| RA | HD2CM539 | HD2SCR | 12 | 6.17e-01 |
| RA | EVA4 | HD2SCR | 12 | 7.91e-03 |
